# The relationship between nurses’ moral competency and missed nursing care: a descriptive-correlational study

**DOI:** 10.1186/s12912-024-02058-w

**Published:** 2024-06-06

**Authors:** Amir Mohamad Nazari, Fariba Borhani, Akbar Zare-Kaseb, Niloofar Zafarnia

**Affiliations:** 1 Student Research Committee, Department of Medical-Surgical Nursing, School of Nursing and Midwifery, Shahid Beheshti University of Medical Sciences, Tehran, Iran; 2 Department of Medical-Surgical Nursing, School of Nursing and Midwifery, Shahid Beheshti University of Medical Sciences, Tehran, Iran; 3 Educational Development Center, Kerman University of Medical Sciences, Kerman, Iran

**Keywords:** Moral competence, Missed nursing care, Nurses, Nursing care

## Abstract

**Background:**

When any aspect of patient care is overlooked or delayed, it is known as Missed Nursing Care (MNC), leading to adverse events such as medication errors, infections, increased mortality rates, and poor prognosis. Moral competence is crucial for clinical nurses as it guarantees high-quality patient care in nursing practice. Thus, this study aimed to investigate the correlation between moral competencies and MNC among nurses.

**Methods:**

This study was conducted with a descriptive-correlational design. The participants in the study were nurses who were currently enrolled at Shahid Beheshti University of Medical Sciences. In order to recruit nurses for the study, a convenience sampling method was implemented. The study tools were completed by a total of two hundred nurses. Research tools included a demographic questionnaire, the Moral Competence of Clinical Nurses Questionnaire, and the Kalisch and Williams Missed Nursing Care (MISSCARE) survey.

**Ethical consideration:**

This study was approved by the Medical Ethics and Law Research Center of Shahid Beheshti University of Medical Sciences.

**Results:**

The mean scores of moral competencies and MNC were 151.83 ± 12.60 and 42.71 ± 9.38, respectively. In other words, descriptive statistics showed that the moral competence score was more than 75%, and the MNC score was less than 50%. Also, there was a significant negative correlation between the total scores of moral competencies and MNC (*r* = -0.38, *p* < 0.001), indicating that more moral competence was correlated with lower levels of MNC.

**Conclusion:**

The study revealed a negative correlation between nurses’ moral competence and MNC, suggesting that enhancing moral competence could reduce MNC. To reduce MNC occurrences, hospitals, and organizations should prioritize moral competency, according to our research.

## Background

Ethical and moral competence are considered synonymous since both theories involve right and wrong actions [1]. In nursing literature, the concepts are frequently used interchangeably, although there is also a distinction between these terms [1, 2]. Moral competence addresses problems and conflicts based on personal moral principles, not societal expectations [3, 4]. Moral competence directly affects a person’s ethical performance, professional nursing competence, patient safety, and patient outcomes in nursing practice [5, 6]. Ethical conduct is crucial in the nursing profession, meaning that nurses must possess ethical skills, sensitivity, motivations, behavior, attitudes, and knowledge to effectively handle ethical dilemmas in healthcare settings [5–11]. Ethically competent nurses prioritizing patient advocacy and respectful nursing practices, can enhance patient satisfaction and improve outcomes [12, 13].

Limited funding, inadequate staffing and resources, and strained nurse-patient relationships increase pressure on nurses, making it challenging to meet all their nursing responsibilities [14]. It is common for nurses to postpone or eliminate specific nursing measures to prioritize critical tasks, resulting in neglected care. Per Kalisch, Missed Nursing Care (MNC) is the term used to describe the neglect or deferment (partially or completely) of any aspect of patient care [15]. According to studies, a concerning percentage of nursing staff, between 47.8% and 98%, failed to finish necessary tasks, putting patients and the institution at risk [16–19]. MNC has been associated with adverse events like medication errors, infections, and higher mortality rates [19–22]. This ultimately leads to lower job satisfaction and increased nurse turnover, resulting in a decline in the quality of nursing service [15, 23].

By addressing the identified predictors of MNC, nurse managers can effectively establish suitable interventions to support the professional role of nurses and ensure the delivery of complete, safe, and quality nursing care [24]. Nevertheless, limited research has been conducted on the associated factors and predictors of MNC. The studies’ findings demonstrate that MNC can be predicted by factors such as the size of hospital facilities, levels of nurse staffing, and patient safety culture [24–26]. In the literature review, we did not find any study that assessed the correlation between the two constructs of moral competence and MNC in nurses. However, the results of a study showed a relationship between moral sensitivity and MNCs [27]. As we know, moral sensitivity is one of the components of moral decision-making, one of the characteristics of a morally competent person in nursing [13]. Therefore, there seems to be a relationship between moral competence and MNCs. If a correlation existed between these constructs, integrating moral competence into educational programs for nurses could reduce MNC and its harmful effects. So, this study aims to investigate the relationship between moral competence and MNC.

## Methods

### Research design

A descriptive-correlational study design was recruited for this research.

### Research setting

The study was conducted in hospitals affiliated with Shahid Beheshti University of Medical Sciences (Ayatollah Taleghani, Imam Hossein, Luqman Hakim, Shohada Tajrish, Mofid and Masih Daneshvari) from July 2023 to February 2024. Convenient sampling was employed to recruit the sample of nurses.

### Research participants

The inclusion criteria were: (1) nurses having at least a BSc degree; (2) willingness and consent to participate in the research; (3) having at least 1 year of clinical practice background. Failure to return the questionnaires or fill them out incompletely was considered the exclusion criterion.

### Data collection

After receiving permission and coordinating with the hospitals, the researcher explained the study goals, received verbal consent, and guaranteed confidentiality for the nurses who consented and met the inclusion criteria. After explaining how to answer the questionnaires to nurses, the researcher gave them questionnaires placed in envelopes and asked them to complete them. A link to the questionnaire was provided to nurses via WhatsApp, Telegram, and email because some participants did not have time or the desire to complete it. Participants were asked to complete the questionnaire at the appropriate time and accurately.

### Research instruments

The research tools consisted of a demographic questionnaire, a Moral Competence of Clinical Nurses questionnaire [13], and the Kalisch and Williams Missed Nursing Care (MISSCARE) survey [28].

### The moral competence of clinical nurses’ questionnaire

The moral competence of clinical nurses’ questionnaire has forty-six items and comprises six dimensions: responsible behavior, client-centric, efficacy, trust capability, desire to serve, and moral knowledge. Each item scored on a 4-point Likert from 1 (Never) to 4 (Always). The total scores range from 46 to 184, with higher scores indicating higher moral competence. This questionnaire’s design and psychometrics properties have been evaluated in a study by Zafarnia et al. The results of the data analysis in the quantitative section of their study showed that the moral competence of clinical nurses’ questionnaires has acceptable face and content validity (S-CVI = 0.92). Also, This questionnaire had adequate internal consistency (α = 0.93) and external stability (ICC = 0.84) [13].

### MISSCARE survey

Kalisch and Williams (2009) developed the psychometrically assessed MISSCARE Survey, which includes 24 items focusing on specific nursing care areas. This questionnaire is made up of four subscales, including “care intervention with the ongoing assessment,” “intervention for individual needs,” “basic care intervention,” and “discharge planning and patient education.” All items of this survey are scored on a 5-point Likert scale from “Never = 0” to “Always = 4”. The survey’s overall score varies from 24 to 96, indicating that a higher score corresponds to greater missed nursing care. The overall score determines the classification of MNC into three levels: low (less than 60%), medium (60–75%), and high (more than 75%) [29, 30]. A Cronbach’s alpha coefficient of 0.93 was calculated for the Persian version of this questionnaire [31].

### Data analysis

We analyzed all data with IBM SPSS Statistics for Windows, version 20 (IBM Corp., Armonk, N.Y., USA). Statistical analyses were performed using frequency, percentages, means, and standard deviations. The relationship between the two main variables was examined using Pearson’s correlation coefficient. In addition, we conducted an Independent Samples T-test to determine the correlation between two main variables and demographic characteristics. All statistical tests were considered significant if the *p*-value was less than 0.05.

### Ethical considerations

The Medical Ethics and Law Research Center of Shahid Beheshti University of Medical Sciences approved this study. Informed written consent was obtained from all subjects or their legal guardian and they were also assured about the confidentiality of their information.

## Results

### Participants

**Fig. 1:**
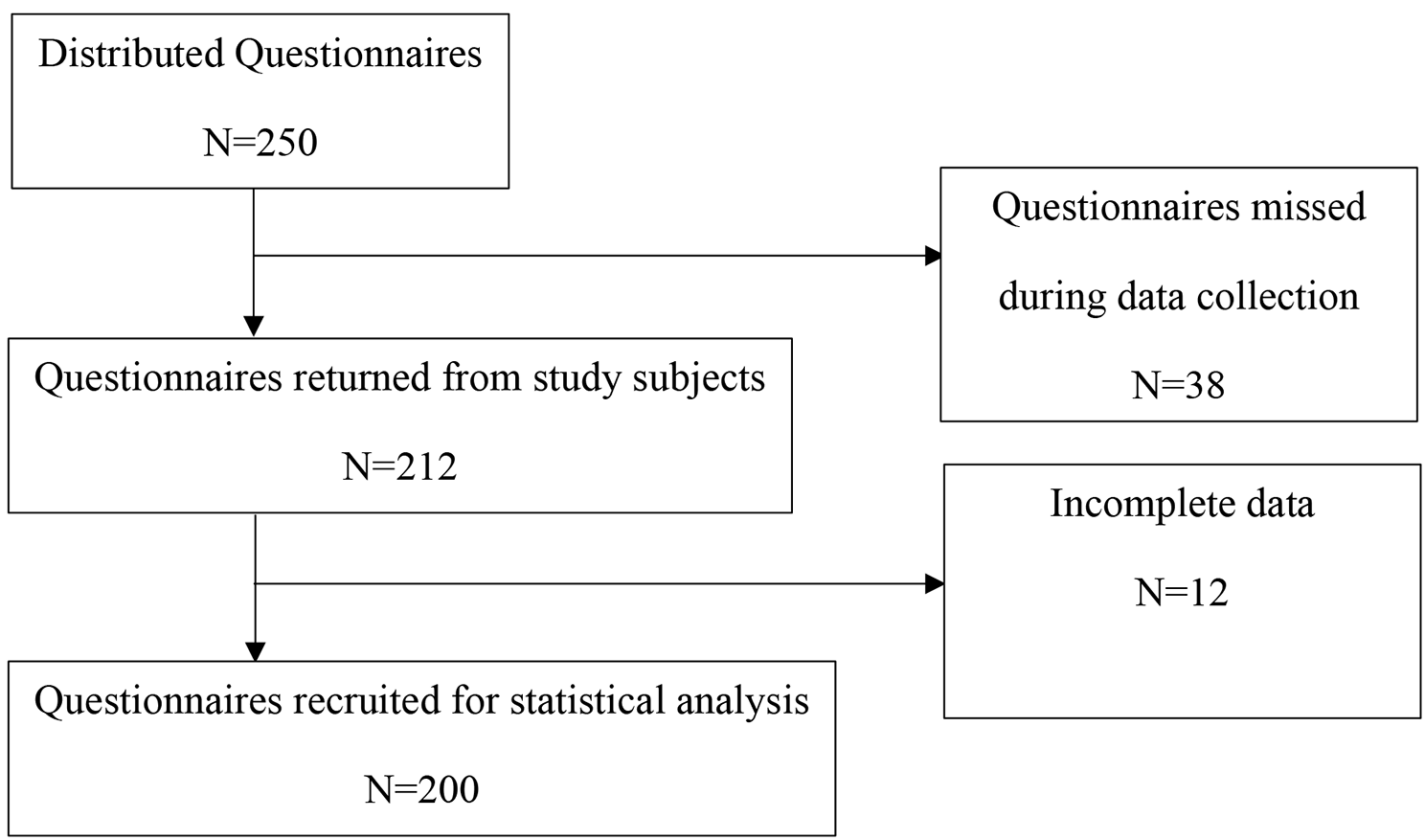
Flow diagram for data collection processes

Two hundred and twelve (84.8%) of the 250 distributed questionnaires were returned from study subjects. After assessing the completed questionnaires, twelve were incomplete and missed demographic data. Finally, two hundred data were analyzed. (Fig. 1.)

### Sample profile

The respondents’ socio-demographic characteristics are summarized in Table 1. The majority of nurses participating in this study were women (71%), less than 30 years old (67%), and had less than five years of work experience (60%). Also, most had a bachelor’s degree (90%) and were married (52%).

**Table 1:** Demographic characteristics (*N* = 200)

Variable		Total (%)
Gender, n (%)	Male	58 (29%)
	Female	142 (71%)
	Total	200 (100%)
Marital status, n (%)	Single	96 (48%)
	Married	104 (52%)
	Total	200 (100%)
Age (years), n (%)	≤ 30	134 (67%)
	> 30	66 (33%)
	Total	200 (100%)
Years of working (year), n (%)	< 5	120 (60%)
	5–10	80 (40%)
	Total	200 (100%)
Education level, n (%)	Bachelor degree	180 (90%)
	Master or above	20 (10%)
	Total	200 (100%)

### Moral competence and MNC

The mean scores for moral competence and MNC were (respectively) 151.83 and 42.71 (Table 2). In other words, descriptive statistics showed that the moral competence score was more than 75%, and the MNC score was less than 50%. Also, there was a significant negative correlation between the total scores of moral competencies and MNC (*r* = -0.38, *p* < 0.001). More moral competence was correlated with lower levels of MNC.

**Table 2:** Descriptive statistics for major study variables

Questionnaire		Range	Mean (SD)	Mean (SD) %
Moral Competence		112–179	151.83 (12.60)	76.69 (9.13)
MNC		24–85	42.71 (9.38)	44.49 (13.02)

### The correlation between the demographic characteristics and major study variables

Another aim of this study was to determine the correlation between the main variables and demographic factors. The independent t-test showed no significant correlation between moral competence and demographic variables. About MNC, a significant correlation with age was also found. So, the mean score was higher in people under 30 (*p* = 0.013). Also, regarding other demographic variables, no significant correlation was found with the mean score of MNC (*p* > 0.05). (Table 3.)

**Table 3:** The correlation between demographic characteristics and major study variables

Demographics	Moral competence		MNC	
	M (SD)	p*	M (SD)	p*
Age		0.888		0.013*
≤ 30	151.75 (13.27)		43.76 (10.04)	
> 30	152.01 (11.20)		40.58 (7.50)	
Gender		0.845		0.290
Male	152.10 (13.56)		43.81 (8.65)	
Female	151.17 (12.23)		42.26 (9.65)	
Marital status		0.684		0.938
Single	151.45 (13.90)		42.65 (9.50)	
Married	152.18 (11.32)		42.76 (9.31)	
Years of working		0.684		0.938
< 5	151.47 (13.53)		43.63 (10.32)	
5–10	152.37 (11.12)		41.32 (7.61)	
Education level		0.086		0.649
Bachelor degree	151.45 (12.95)		42.81 (9.09)	
Master or above	155.20 (8.38)		41.80 (11.90)	

### Descriptive statistics for the dimensions of the moral competence questionnaire

Descriptive Statistics showed that the Moral competence score was over 70% in all questionnaire dimensions. (Table 4.)

**Table 4:** Descriptive statistics for the dimensions of the moral competence questionnaire

Dimensions	Range	Mean (SD)	Mean (SD) %
Responsible Behavior	34–60	50.37 (4.42)	78/60 (9/82)
Trust Capability	18–32	27.30 (2.60)	80/42 (10/83)
Desire to Serve	8–20	15.65 (2.61)	71/01 (17/33)
Efficacy	11–20	16.60 (1.95)	77/33 (13/01)
Client-centric	20–36	28.80 (3.10)	73/33 (11/48)
Moral Knowledge	8–16	13.10 (1.76)	75/83 (14/66)

### The correlation between moral competence dimensions and the MNC score

Pearson’s correlation results showed that the correlation between the score of the responsible behavior dimension and MNC was − 0.38 and was statistically significant (*p* < 0.001). As the responsible behavior score increases, the rate of MNC decreases. Correlation in other dimensions was statistically significant but weak. (Table 5.)

**Table 5:** The correlation between moral competence dimensions and the MNC score

	Responsible Behavior	Trust Capability	Desire to Serve	Efficacy	Client-centric	Moral Knowledge
Pearson Correlation Coefficient	− 0.378^**^	− 0.225^**^	− 0.201^**^	− 0.268^**^	− 0.360^**^	− 0.210^**^
*P* value	0.000	0.001	0.004	0.000	0.000	0.003

### The correlation between demographic characteristics and moral competence dimensions

The independent t-test results showed no significant relationship between the score of moral competence dimensions and age, gender, and marital status (*p* > 0.05). However, the mean scores of client-centric dimensions were higher in nurses who had experience of 5 to 10 years (*p* = 0.018) and higher education level (*p* = 0.021). Still, the other dimensions had no statistically significant differences (*p* > 0.05).

## Discussion

This study investigated the correlation between moral competency and nurses’ MNC level. Results showed a significant correlation between moral competency and nurses’ MNC level. More moral competency was correlated with lower levels of MNC. Also, MNC has been significantly related to the responsible behavior dimension of nursing moral competence dimensions. This was consistent with a cross-sectional study’s findings that higher personal accountability was significantly associated with reduced MNC [32].

In the existing literature, personal responsibility, professional responsibility and responsibility towards the client are all considered to be required to provide moral care in clinical practice [33]. As we know, moral care is one of the main structures of moral competence [13]. According to a study, a strong sense of personal responsibility was found to be significantly linked to a reduced occurrence of MNC [34]. There seems to be such a justification behind the relationship between moral competence and MNC.

Many studies have examined the moral competence and MNC, separately. The correlation between moral competence and MNCs has not been analyzed.

### Moral competence

The results of our investigation demonstrate that nurses in the studied society possess a commendable level of moral competence. Although studies in this field are limited, one study revealed that nursing leaders exhibit a significant level of moral competence [35].

Our study found no significant relationship between demographic variables and moral competence. The findings of our study align with those of a recent study on this matter. The results of the mentioned study have shown that with any of the demographic characteristics, a clinical nurse could have high or low ethical competence [36]. A recent review study has categorized the factors impacting nurses’ moral competence into two groups: promoting factors and barriers. Multidisciplinary cooperation, effective leadership, communication, ethical consultation, creativity, and intuition were all promoting factors. Barriers also included overwhelming clinical situations, lack of organizational support, workload pressure, insufficient resources, lack of written policies, knowledge, experience, and ethical awareness [8].

One’s moral competency influences many outcomes. Clinical nurses’ moral competence in nursing promotes critical thinking, issue analysis, ethical decision-making, problem-solving, and ethical behavior in their daily work. Thus, moral competence promotes high-quality care [8, 35, 36].

### Missed nursing care

The existing literature has not discussed the relationship between moral competence and MNC. MNC was strongly linked to moral competence, as shown in our study. MNC is only associated with nurses’ age, suggesting older nurses have lower rates of missed care. Just like our study, a descriptive study conducted in Iran examined factors linked to MNCs, including age as one of the factors. In contrast to our research, this study also identified the number of patients under care and teamwork as correlated factors [37].

Nevertheless, the outcomes of the two studies have shown a significant correlation between gender and the extent of MNC. This case was more prevalent among male nurses [38, 39]. Furthermore, one of the studies revealed that individuals who worked extended hours and experienced overtime and those who perceived the nurse-to-patient ratio as insufficient exhibited higher MNC scores [38].

The results of a systematic review stated that MNC is not related to any of the variables such as country, type of hospital, location, gender, age, experience, or education of nurses. This aligns with our findings to some extent, except that age in our study had a significant relationship with MNC [40].

Current literature emphasizes staffing levels and labor resources, nurses’ skills, insufficient material resources, patient safety culture, caring behaviors, patient acuity, teamwork, and communication as factors associated with MNC [40–42]. MNC also leads to poorer overall quality of patient care, poorer patient satisfaction, poorer nurses’ job satisfaction, higher intent to leave, increased patient adverse events, hospital length of stay, and increased hospital readmission [26, 40, 43].

The consequences of MNCs highlight the necessity of identifying their influencing factors. MNC decreases with improved moral competence in nurses. Therefore, programs that can nurture moral competence in nurses should be considered [44]. Findings from a study exploring ethics education in nursing revealed that involving students in case study discussions and utilizing ethical frameworks can enhance students’ moral competence despite the absence of strong evidence [45].

Another study in this field discovered that incorporating ethical discussions in health care can enhance professionals’ moral competence and the quality of care they provide [46]. Other studies have been conducted to develop moral competence in nursing students. A longitudinal study found that ethics education can improve students’ moral competence [2]. Nevertheless, nurse educators face challenges when trying to improve students’ ethical competence in clinical settings. These challenges encompassed the disparity between theoretical instruction and clinical application, scarcity of adequate role models, and insufficient resources [47].

Finally, it seems that increasing the moral competence of nurses can be a practical solution in reducing MNCs and thus improving the quality of care. However, future research endeavors should place greater emphasis on the investigation of effective pedagogical methods for developing moral competence.

### Strength and limitations

The correlation between these two constructs has never been studied before. We are the first to examine the relationship between these two concepts in our study. The inability to prove causation is a limitation in correlational studies like this. Participants were exclusively recruited from a single city, which limited the study. Cultural differences can influence the main variables of this study. Thus, the relationship between MNC and the moral competence of nurses cannot be adequately explained by considering only one city. To enhance generalizability, it would be advantageous to include more cities.

### Implications for nursing education and practice

The common clinical challenge of MNCs has been a recent concern for hospitals. Numerous factors can make this situation challenging. Improved nursing performance has resulted from studying the various causes. Low moral competence is a factor we found that can increase MNC. Numerous approaches can be employed to enhance the moral competence of nurses. To accomplish this goal, we can introduce alterations to the educational curriculum, plan nursing ethics workshops, host nursing ethics events, encourage discussions, and integrate nursing ethics into clinical rounds.

## Conclusion

The data gathered from our study strongly suggests that nurses in the society being studied possess a praiseworthy level of moral competence. Nurses also reported low levels of MNC. Lower levels of MNCs were associated with higher moral competency. While there was no significant relationship between moral competence and demographic variables, it was found that the age of the nurses had a significant impact on the MNC. Our research indicates the importance of moral competency in decreasing MNCs’ occurrence in clinical settings. The emphasis of further research should be on interventions that target the enhancement of nurses’ moral competency and mitigating MNC.

## Data Availability

The datasets used and/or analyzed during the current study are available from the corresponding author on reasonable request.
